# Dietary factors and risk of mortality among patients with esophageal cancer: a systematic review

**DOI:** 10.1186/s12885-020-06767-8

**Published:** 2020-04-06

**Authors:** Li-Ping Sun, Lu-Bin Yan, Zhen-Zhen Liu, Wen-Jing Zhao, Cai-Xia Zhang, Yu-Min Chen, Xiang Qian Lao, Xudong Liu

**Affiliations:** 1 Department of Epidemiology, School of Public Health, Sun Yat-sen University, Guangzhou, China; 2 Department of Pediatric Surgery, the Sixth Affiliated Hospital, Sun Yat-sen University, Guangzhou, China; 3 Department of Radiology, the Third Affiliated Hospital, Sun Yat-sen University, Guangzhou, China; 4 Department of Public Health, Faculty of Medicine, Hokkaido University, Sapporo, Japan; 5 JC School of Public Health and Primary care, the Chinese University of Hong Kong, Hong Kong, China

**Keywords:** Dietary intake, Esophageal Cancer, Mortality, Systematic review, Meta-analysis

## Abstract

**Background:**

The effects of dietary factors on prognosis of esophageal cancer remain unclear. This systematic review and meta-analysis aimed to assess the association between dietary intake and the risk of mortality among patients with esophageal cancer.

**Methods:**

Six electronic databases (PubMed, Web of Science, OVID, ProQuest, CNKI and Wanfang) were searched for studies published up to Oct. 2019 that examined the association between dietary intake and all-cause mortality, esophageal cancer-specific mortality and esophageal cancer recurrence. The pooled hazard ratio (HR) with 95% confidence interval (CI) were derived by comparing the highest with the lowest categories of each dietary item and by using random effect models.

**Results:**

A total of 15 cohort studies were included in this study and all reported pre-diagnosis dietary exposure; two focused on dietary folate, 12 on alcohol consumption and three on other dietary components (sugary beverages, phytochemicals and preserved vegetables). When comparing the highest with the lowest categories, dietary folate intake was associated with a reduced risk of esophageal cancer-specific mortality in patients with esophageal squamous cell carcinoma (HR: 0.41, 95% CI: 0.25–0.69), with low heterogeneity (*I*^2^ = 0%, *P* = 0.788). When comparing the highest with the lowest categories of alcohol consumption, alcohol consumption was associated with an increased risk of all-cause mortality in patients with esophageal squamous cell carcinoma (HR: 1.29, 95% CI: 1.07–1.55; heterogeneity: *I*^2^ = 53%, *P* = 0.030), but this increased risk was not significant in patients with esophageal adenocarcinoma (HR = 1.05, 95% CI: 0.84–1.32).

**Conclusions:**

This review with pre-diagnostic dietary exposure showed that dietary folate intake was associated with a reduced risk of mortality of esophageal squamous cell carcinoma, whereas alcohol consumption was associated with an increased risk. More studies are needed to investigate effect of dietary factors, especially post-diagnosis dietary consumption, on esophageal cancer prognosis.

## Background

Esophageal cancer (EC) is one of the most malignant tumors worldwide, ranking seventh in cancer incidence and sixth in cancer mortality in 2018 [1]. Esophageal adenocarcinoma (EAC) and esophageal squamous cell carcinoma (ESCC) are two main histological types. EAC is the main histological type in developed countries whereas ESCC predominates in eastern Asia and Africa [2]. The prognosis of esophageal cancer is poor; the 5-year survival rate of EC in the United States is 19%, in Europe 12.4%, and in China 20.9% [3–5].

The prognosis of esophageal cancer is influenced by many different factors [6]. Noteworthy, increasing evidence is highlighting the pivotal effects of nutritional factors on cancer prognosis and survival: natural ingredients such as lycopene and beta-carotene in the plant food could inhibit EC109 cell viability [7], dietary interventions could improve diet quality in cancer survivors [8], and nutritional support could improve esophageal cancer prognosis by improving treatment compliance, reducing toxicity and enhancing the immune response [9]. However, most studies focused on the roles of perioperative nutrition support in postoperative complications [10, 11], and the results from epidemiological studies on the associations between dietary factors and EC prognosis are inconclusive [12–15]. A meta-analysis showed that pre-diagnosis alcohol drinking increased risk of death in ESCC rather than in EAC [16], however, some new evidence has emerged after this study published and the pooled results are needed to be updated.

Therefore, by summarizing the results of observational studies, this systematic review and meta-analysis was conducted to evaluate the association between food and its components intake and risk of mortality among patients with esophageal cancer, with addressing the difference in histology and the difference between all-cause mortality and cancer-specific mortality.

## Methods

### Literature search

Literatures published up to October. 2019 were systematically searched through four English databases (PubMed, Web of Science, OVID, ProQuest) and two Chinese databases (CNKI and Wanfang). PRISMA statement for conducting and reporting meta-analysis of observational studies was followed [17]. The search strategy was as follows: [(vegetables OR fruits OR meat OR poultry OR drinking OR alcohol OR beer OR liquor OR beverage OR nuts OR soy OR cereal OR bean OR nutrients OR micronutrients OR macronutrients OR dietary fiber OR vitamin OR phytochemicals OR lignan OR phytoestrogen OR dietary OR diet OR food OR dietary pattern OR dietary supplements)] AND [(esophageal cancer OR esophageal adenocarcinoma OR esophageal squamous cell carcinoma OR esophageal neoplasm OR esophageal tumor OR cancer of esophagus OR esophageal neoplasms)] AND [(Survival OR prognosis OR mortality OR recurrence OR replase OR progression OR medical futility OR treatment outcome OR treatment failure OR cause of death OR fatal outcome)]. Language and countries were not restricted during the whole searching process.

Two researchers (LP Sun & LB Yan) independently conducted the literature retrieval, identified potential studies, extracted information from the included papers, and assessed the quality of included studies. Discrepancies were settled down by group discussion with other two professionals (X LIU & CX Zhang). The titles and abstracts of initially identified papers were firstly reviewed, and then the full texts of the selected papers were reviewed to determine eligibility. To avoid omission of literatures, backward and forward citation tracking in both Web of Science and Scopus were also used to identify articles.

### Study selection criteria

Studies meeting the following conditions would be included: (i) study design was cohort study with esophageal cancer patients; (ii) the consumption of food and/or its components but not supplementations was accessible; (iii) prognostic outcomes included all-cause mortality, esophageal cancer-specific mortality or esophageal cancer recurrence; (iv) the hazard ratio (HR) or relative risk with 95% confidence interval (95% CI) were reported or could be calculated. When there were papers from the same study or covering the same population, only the most comprehensive or latest data was selected. Case reports, cross-sectional studies, editorials, abstracts, reviews, articles without full text, duplicated studies, animal studies and vitro studies were excluded.

### Data extraction

Data and information were extracted from the included studies, including name of the first author, publication year, country, sources of patients, sample size, histological type, follow-up duration, stage/grade grouping, dietary exposure, dietary assessments, outcomes, comparison method, effect size, confounders and covariates.

### Quality assessment

Quality of the included papers was evaluated according to the Newcastle-Ottawa Scale (NOS) Criteria for non-randomized studies [18]. A maximum total 9 points were assigned to each study, with a maximum of 4 for selection, 2 for comparability and 3 for outcome. Studies were considered to be of high quality (> 6), median quality (4~6) or low quality (≤3, [19]).

### Statistical analyses

Meta-analysis was conducted to estimate the pooled HR with 95% CI by comparing the highest with the lowest categories of dietary intake in each selected item. A random effect model was selected in the meta-analysis [20]. Heterogeneity between studies was measured using *Q* and *I*^2^ statistics. Sensitivity analysis was conducted by excluding the study one by one from the pooled results, by excluding the studies not reporting adjusted effects, and by excluding the studies not collecting information of stage and severity of esophageal cancer. The meta-analysis with crude HRs obtained from univariate analysis was also conducted. Publication bias was tested by funnel plot with Begg’s tests [21]. A power calculator used to estimate statistical power of meta-analyses [22]. Statistical analysis was completed in Stata 15.1 (Stata Corporation, College Station, TX) and R software (version 3.5.3).

## Results

### Literature retrieval

**Fig. 1:**
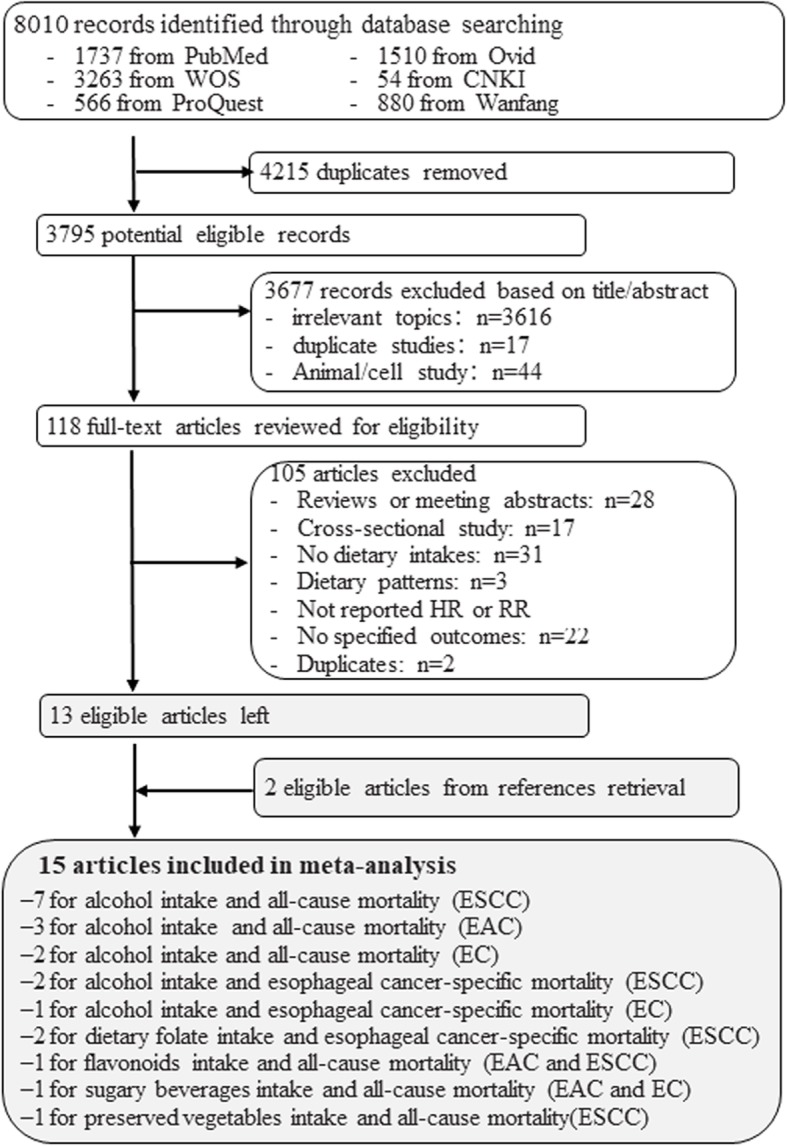
Flowchart of the literature selection. Abbreviation: WOS, Web of Science; HR, hazard ratio; RR, relative risk; OR, odd ratio; EC, esophageal cancer; ESCC, esophageal squamous cell carcinoma; EAC, esophageal adenocarcinoma. CNKI and Wanfang are Chinese database, CNKI: http://new.oversea.cnki.net/index/; Wanfang: http://www.wanfangdata.com.cn/resource_nav/index.do

Figure 1 shows the flowchart of literature selection. After removal of 4215 duplicates, 3795 potentially eligible records were left, of which 3677 were excluded after reviewing the titles and abstracts. Finally, 15 eligible cohort studies [12–15, 23–33] containing 6826 esophageal cancer patients were identified through full text review of 118 studies. The most common reasons for exclusion were study designs, lack of data on dietary exposure and no outcomes of interest.

### Characteristics of included studies

Characteristics and study quality of the included studies are shown in Table 1. Briefly, 7 cohort studies recruited patients from completed case-control studies [12–14, 23, 29, 31, 32] and the other eight were new established cohorts [15, 24–28, 30, 33]; three studies were conducted in USA [12, 13, 23], six in China [15, 24, 25, 27, 30, 32], two in Australia [29, 31], one in South Korea [28], one in Sweden [14], one in Japan [26] and one in Iran [33]; one study only recruited male patients [28] and the others included both genders. The median follow-up duration ranged from 0.8 to 12.1 years. Only three studies reported the risk estimates without any adjustments [23–25], other 12 studies reported adjusted risk estimates. The most common adjusted confounders included age, gender, tumor stage, complications and treatments. With the exception of five studies [23–25, 28, 30], other ten studies collected information of stage and severity of esophageal cancer and adjusted for them when estimating the effect size. Eleven studies focused on ESCC [13–15, 23–27, 29, 30, 32], 5 on EAC [12–14, 23, 31], and 3 on EC [12, 28, 33].

**Table 1:** Characteristics of included studies and quality score

Author, reference, year, country	Sources of cohort patients	Follow-up duration (years)	Number and type of patients	Stage/grade grouping	Exposure	Dietary assessments	Comparison categories	Adjusted HR (95% CI) (highest vs. lowest) for mortality	Adjustments	NOS stars
Petrick et al. [13], 2015, USA	Population-based case–control study	Max: 7.5 Median^a^: 0.8 for EAC, 0.9 for ESCC	274 EAC 191 ESCC	Primary invasive cases: localized, regional, distant, unknown	Total flavonoids, six classes of flavonoids (anthocyanidins, flavan-3-ols, flavanones, flavones, flavanols, and isoflavones), lignans	A 104-item modified FFQ	Each were divided into 4 categories (mg/day): Total flavonoids: 0–62.35, 62.36–103.39, 103.40–253.24, ≥253.25; Anthocyanidins: 0–6.23, 6.24–10.11, 10.12–16.23, ≥16.24; Flavan-3-ols: 0–10.90, 10.91–26.67, 26.68–210.51, ≥210.52; Flavanones: 0–8.63, 8.64–32.94, 34.95–49.00, ≥49.01; Flavones: 0–1.20, 1.21–1.81, 1.82–2.64, ≥2.65; Flavonols: 0–8.16, 8.17–12.30, 12.31–19.34, ≥19.35; Isoflavones: 0–0.31, 0.32–0.46, 0.47–0.62, ≥0.63; Lignans: 0–0.044, 0.045–0.060, 0.061–0.079, ≥0.080	All-cause mortality For EAC: Total flavonoids: 0.98 (0.68, 1.41); Anthocyanidins: 0.87 (0.60, 1.26); Flavan-3-ols: 0.93 (0.65, 1.33); Flavanones: 1.15 (0.79, 1.68); Flavones: 0.83 (0.58, 1.19); Flavonols: 0.94 (0.65, 1.37); Isoflavones: 0.75 (0.49, 1.13); Lignans: 0.78 (0.54, 1.14) For ESCC: Total flavonoids: 0.91 (0.58, 1.44); Anthocyanidins: 2.272 (0.66, 1.56); Flavan-3-ols: 1.09 (0.69, 1.74); Flavanones: 1.24 (0.76, 2.03); Flavones: 2.272 (0.64, 1.54); Flavonols: 0.93 (0.61, 1.40); Isoflavones: 0.97 (0.60, 1.58); Lignans: 0.61 (0.39, 0.96)	Cancer stage and dietary energy intake	7
Miles et al. [12], 2016, USA	Population-based case–control study	Median: 12.1	108 EC 74 EAC	Well differentiated, Poorly differentiated, Undetermined	Sugary beverages including soft drinks and fruit juices (classified into SB1 and SB2^b^)	NCI-block FFQ	Median for soft drinks and fruit juices(g/day): soft drinks:4.0; fruit juices:0.71 SB1 intake quartile point(g/day): (Q1)0.71, (Q2)11.81, (Q3) 40.00; SB2 intake quartile points(g/day) (Q1)3.04, (Q2)20.76, (Q3)45.29	All-cause mortality For EC: soft drinks: 2.29 (1.32,3.93); fruit juices: 2.39 (1.34,4.30); SB1: 2.58 (1.45,4.60); SB2: 1.94 (1.06,3.53) For EAC: soft drinks: 1.84 (0.92,3.68); fruit juices: 1.60 (0.79,3.25); SB1: 1.51 (0.72,3.16); SB2: 1.44 (0.57,3.62)	Age, sex, ethnicity, education, smoking, alcohol drinking, caloric intake, pathology type, and tumor differentiation grade	8
Shi et al. [30], 2018, China	A new established patient cohort	Median: 4.08	185 ESCC	AJCC stage: Only included I and II stage	Preserved vegetables	A modified FFQ	2 categories (time/week): < 1 and ≥ 1	All-cause mortality 1.58 (1.01,2.47)	Age and sex	7
Lu et al. [24], 2011, China	A new established patient cohort	Median: 3.5, (Min-Max: 0.03–4.66)	120 ESCC patients underwent esophagectomy	T stage: T1~T4 N stage: N0, N1 M stage: M0, M1 clinical stage: 1/2, 3/4	Folate	NIH-modified FFQ	3 categories(ug/day): < 30.0, 30.0–95.4, ≥95.5	Cancer-specific mortality 0.39 (0.20,0.78)	Age, sex, TNM stage	7
Jing et al. [25], 2012, China	A new established patient cohort	Median: 3.25, (Min-Max: 0.25–5)	167 ESCC	T stage: T1~T4 N stage: N0, N1 M stage: M0, M1	Folate	A 65-item self-administered structured questionnaire	3 categories(ug/day): < 230, 230–300, > 300	Cancer-specific mortality: 0.45 (0.18,0.87)	Age, sex, smoking, drinking, tumor sites, TNM stage, chemo-therapy and radio-therapy	6
Trivers et al. [23], 2005, USA	population- based, case-control study	Max: 7.5 Median ^a^: 0.8 for EAC and 0.9 for ESCC	293 EAC 220 ESCC	Incident invasive cases: localized, regional, distant, unknown	Alcohol	Baseline interviews	2 categories: non-drinkers and ever drinkers ^c^	All-cause mortality ^d^: EAC: 1.08 (0.81,1.44) ESCC: 1.77 (0.93,3.35)	None	7
Park et al. [28], 2006, South Korea	A cohort of male participated in a national health examination program	Mean:2.05 (Max:6.8)	272 EC	Not collected	Alcohol	A self-administered questionnaire	3 categories(g of alcohol/week): 0, 0–124.1, ≥124.2	All-cause mortality 1.44 (0.81,2.55)	Age, BMI, fasting serum glucose level, cholesterol level, physical activity, food preference, blood pressure, and other comorbidities	5
Samadi et al. [33], 2007, Iran	Patients initially diagnosed in Aras Clinic	Max: 5	122 EC	Differentiation:well, moderate/ poor, nondifferentiated	Alcohol	Questionnaire completed at the time of diagnosis	2 categories: no and yes	All-cause mortality: 7.51(0.82,69.10)	Age, sex, residence, treatment, smoking, opium use, differentiation, education	4
Sundelof et al. [14], 2008, Sweden	Nationwide case-control study	From 1994.12.1–1997.12.31 to 2004.12.31	177 EAC 159 ESCC	TNM stage: I, II, III, IV	Alcohol (including beer, wine and liquor)	Computer-aided face-to-face interview with separate questions	4 categories(g of pure alcohol/week): never, 1–15, 16–70, > 70	All-cause mortality EAC:1.0 (0.5,1.7) ESCC:0.6 (0.3,1.4)	Age, sex, educational level, symptomatic reflux, BMI, smoking, physical activity, tumor stage and for esophagectomy	8
Shitara et al. [26], 2010, Japan	Patients in Aichi Cancer Center Hospital	Median:5.6 (Min-Max: 2.1–7.9)	363 ESCC	UICC stage: I, II, III, IV	Alcohol	HERPACC questionnaire	2 categories^e^ (g of ethanol/week): <230 and ≥ 230	All-cause mortality 0.85(0.61,1.18)	Age, sex, smoking, ECOG PS, tumor length, UICC stage, treatment	7
Lu et al. [24], 2011, China	A new established patient cohort	Median: 3.5, (Min-Max: 0.03–4.66).	120 ESCC patients underwent esophagectomy	T stage: T1~T4 N stage: N0, N1 M stage: M0, M1 clinical stage: 1/2, 3/4	Alcohol	Risk factor questionnaire	2 categories: Never drinkers and ever drinkers	Cancer-specific mortality: 1.02(0.61–1.72)	None	6
Jing et al. [25], 2012, China	Patients in General Hospital of Chengdu Military Area	Median: 3.25 (Min-Max: 0.25–5)	167 ESCC	T stage: T1~T4 N stage: N0, N1 M stage: M0, M1	Alcohol	A 65-item self-administered structured questionnaire	4 categories^f^ (g of ethanol/week): never, former, moderate, heavy	Cancer-specific mortality: 1.42(0.83,1.84)	None	5
Thrift et al. [29], 2012, Australia	Population-based case-control study	Median: 6.4 (Min-Max: 4.8–8.9)	301 ESCC	AJCC stage: I, II, III, IV	Alcohol (including light beer, regular beer, white wine, red wine, port/sherry and spirits/liqueur)	A health and life style questionnaire	4 categories(g of ethanol/week) < 10, 10–60, 70–200, ≥210	All-cause mortality ESCC: 2.08 (1.18,3.69);	Age, sex, AJCC stage, treatment intent, number of comorbidities and smoking	7
Thrift et al. [31], 2012, Australia	Population-based case-control study	Median: 6.4 (Min-Max: 4.8–8.9)	362 EAC	AJCC stage: I, II, III, IV	Alcohol (including light beer, regular beer, white wine, red wine, port/sherry and spirits/liqueur)	A health and life style questionnaire	4 categories(g of ethanol/week) < 10, 10–60, 70–200, ≥210	All-cause mortality EAC: 1.02(0.64,1.64)	Age, sex, AJCC stage, treatment intent, number of comorbidities and smoking	7
Wu et al. [32], 2013, China	Hospital-based case-control study	Max: 5	718 ESCC	AJCC stage: I + II, III + IV	Alcohol	A standardized questionnaire	2 categories^g^: users and non-users	All-cause mortality: 1.30(1.01,1.67);	Age, sex, education levels and AJCC stages	8
Huang et al. [27], 2014, China	Prospectively created esophageal carcinoma database	Median:5.3	2151EC 1851 ESCC,	AJCC stage: 0 + I, II, III	Alcohol (including wine, spirit and beer)	Baseline interviews	4 categories^h^ (g of alcohol/week): 0, 0–90.09, 91–272.09, ≥273	All-cause mortality ^d^: EC: 1.46(1.19,1.79) ESCC: 1.37(1.11,1.70)	Age, sex, weight loss, stage, radicality of surgery, adjuvant treatment, smoking	7
Ma et al. [15], 2016, China	Esophageal cancer database of the Department of Thoracic Surgery at Sun Yat-sen University Cancer Center	Median: 6.5 (Min-Max:1–20)	643 ESCC with negative lymphatic metastasis having undergone esophagectomy	Post-operation staging of AJCC stage: IA, IB, IIA	Alcohol	Medical records	2 categories^i^: non-drinkers and drinkers	All-cause mortality ^d^: 1.58(1.21,2.07)	Age, sex, smoking, family history, tumor location, surgery technique, post-operation staging, tumor grade	7

*HR* hazard ratio, *CI* confidence interval, *NOS* the Newcastle-Ottawa Quality assessment scale, *EC* esophageal cancer, *EAC* esophageal adenocarcinoma, *ESCC* esophageal squamous cell carcinoma, *FFQ* food frequency questionnaire, *NCI* National Cancer Institute, *NIH* National Institutes of Health, *BMI* body mass index, *Min* minimum, *Max* maximum, *TNM* tumor node metastasis, *HERPACC* Hospital-based Epidemiologic Research Program at Aichi Cancer Center, *ECOG PS* Eastern Cooperative Oncology Group performance status, *AJCC* American Joint Committee on Cancer, *UICC* Union for International Cancer Control

^a^ Data of median survival time. Survival time in this article was defined as the time from the date of diagnosis to the date of death or last follow-up, same as the time of follow-up

^b^ SB1, Sugars from soft drinks and fruit juices (g/day); SB2, Sugars from soft drinks, fruit juices, and sugar added to tea, coffee or cereal (g/day)

^c^ Ever drinkers defined as those who had ≥1 alcoholic drink (12 oz. beer, 4 oz. glass of wine, 1 drink with hard liquor) per month for ≥6 months

^d^ The outcome was overall survival (OS) defined as the time from diagnosis through death from any causes. Calculated HR was the same as that of all-cause mortality

^e^ Calculated according to definition of drinking group in this article. It divided alcohol intake into 2 categories: non-heavy drinkers and heavy drinkers

^f^ Former drinkers were those who quit drinking more than 1 year, heavy drinkers were those who drank alcoholic beverages ≥250 g of ethanol/week while moderate drinkers were defined as drinkers consuming < 250 and > 0 g of ethanol/week

^g^ Users were defined as those who consumed alcoholic drinks ≥1 time /week for ≥6 months

^h^ Calculated according to definition of drinking group in this article. It divided alcohol intake into 4 categories: non-drinkers, light drinkers, moderate drinkers and heavy drinkers

^i^ Patients with a present or past history of alcohol consumption were referred to as drinkers

All of 15 included studies provided pre-diagnosis dietary exposure information. In terms of types of dietary exposure, 12 studies were on alcohol consumption [14, 15, 23–29, 31–33], two on dietary folate intake [24, 25], one on sugary beverage [12], one on flavonoids and lignans [13] and one on preserved vegetables [30]. A total of 13 studies [12–15, 23, 26–33] used all-cause mortality as outcome and the other two [24, 25] used esophageal cancer-specific mortality. Consumption of alcohol was measured using health behavior questionnaires, while intakes of dietary folate, flavonoids, preserved vegetables and sugary beverages were collected from validate modified food frequency questionnaire. Of the five studies not collecting information of stage and severity of esophageal cancer, one was focused on preserved vegetables [30] and the other four on alcohol [23–25, 28].

For study quality of 15 included studies, average NOS score was 6.65, ranging from 4 to 8; 12 studies were high quality (NOS score ≥ 7) and three studies were median quality.

### Dietary folate intake

**Fig. 2:**
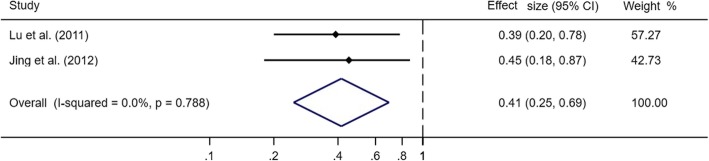
Forest plot of association between dietary folate intake (highest vs. lowest) and esophageal cancer-specific mortality among patients with esophageal squamous cell carcinoma

**Fig. 3:**
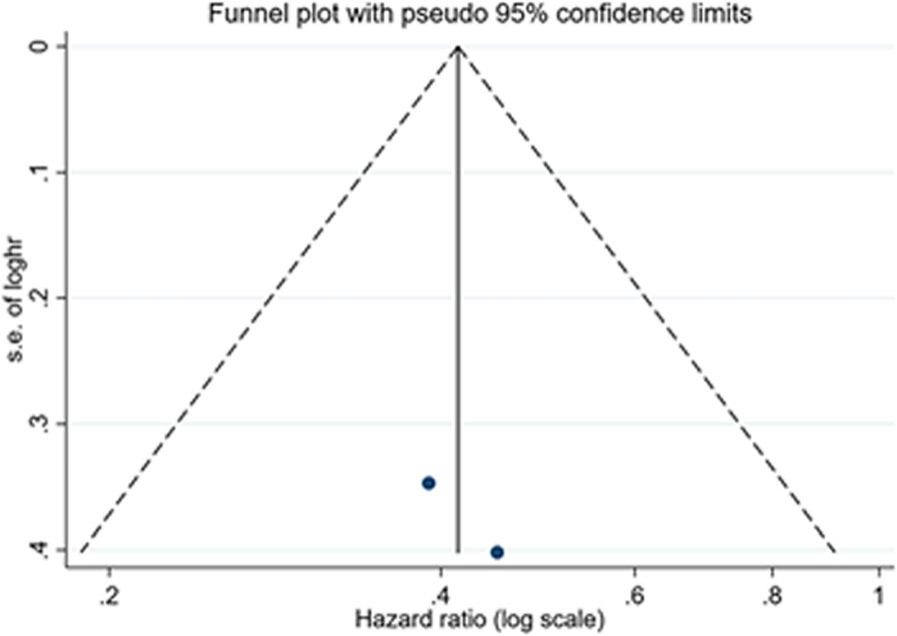
The funnel plot with Pseudo 95% confidence limits on dietary folate intake and esophageal cancer-specific mortality among patients with esophageal squamous cell carcinoma

Only two studies [24, 25] reported effects of dietary folate intake on esophageal cancer-specific mortality in ESCC. These two studies provided adjusted risk estimates from multivariate analysis. Categorization of folate intake was different in these two studies. The highest vs. the lowest in one article [24] was ≥95.5 μg/day vs. < 30.0 μg/day, while in another article [25] was > 300μg/day vs. < 230μg/day. When pooled these two studies (Fig. 2), the overall HR was 0.41 (95% CI: 0.25–0.69) with low statistical heterogeneity (*P*_for heterogeneity_ = 0.79, *I*^2^ = 0%). The funnel plot did not reveal asymmetry (Fig. 3) and the corresponding Begg’s test did not show publication bias (*P* = 1.00). Power calculation for random effect model was 96.5%.

### Other dietary components

Only one study on sugary beverages [12], one on phytochemicals [13] and one on preserved vegetables [30] were found. Miles et al. [12] studied effects of sugary beverages intake on prognosis of EC and EAC. They found that soft drinks and fruit juices intake would worsen prognosis of EC patients; however, when the study population was restricted to patients with EAC, no significant association between sugary beverages and all-cause mortality was found. Petrick et al. [13] studied effects of consumption of total dietary flavonoids, dietary flavonoid subclasses (anthocyanidins, flavan-3-ols, flavanones, flavones, flavonols and isoflavones) and lignans on all-cause mortality in ESCC and EAC, respectively; however, only lignans was found to reduce all-cause mortality of ESCC by 42% (HR = 0.58, 95% CI: 0.37–0.92). As indicated in a study done by Shi et al. [30], in ESCC patients, when comparing with patients consuming preserved vegetables < 1 time/week, those who consumed preserved vegetables ≥1time/week had a 1.58-fold (95% CI: 1.01–2.47) risk of all-cause mortality.

### Alcohol consumption

A total of ten studies [14, 15, 23, 26–29, 31–33] investigated effects of alcohol consumption on all-cause mortality and the other two studies [24, 25] on esophageal cancer-specific mortality (Table 1). The reference group (the lowest group) in nine studies [14, 15, 23–25, 27, 28, 32, 33] was non-drinkers, in two studies [29, 31] was those consuming < 10 g ethanol per week, and in one study [26] was non-heavy drinkers. The highest group was defined as ever drinkers [23, 24], or current drinkers [15, 32, 33], or group with the highest level of alcohol consumption [14, 25–29, 31]. Only five studies [23–26, 29] was available to estimate the risk by using univariate analysis (Table 2).

**Table 2:** Hazard ratios (HRs) with 95% confidence intervals (CIs) for alcohol consumption and mortality of esophageal cancer by histological type

Study ID ^a^	Crude HR (95% CI) ^b^	Adjusted HR (95% CI) ^b^	Heterogeneity for pooled crude HR		Heterogeneity for pooled adjusted HR	
			*I*^2^ (%)	*P*	*I*^2^ (%)	*P*
EC			/	/	4.2	0.352
Huang et al. [27]		1.46 (1.19,1.79)				
Samadi et al. [33]		7.51 (0.82, 69.10)				
Park et al. [28]		1.44 (0.81, 2.55)				
pooled estimates		1.48 (1.19, 1.84)				
ESCC			77.8	0.001	66.9	0.010
Huang et al. [27]		1.37 (1.11, 1.70)				
Ma et al. [15]		1.58 (1.21, 2.07)				
Shitara et al. [26]	0.87 (0.65, 1.17)	0.85 (0.61, 1.18)				
Sundelof et al. [14]		0.60 (0.30, 1.40)				
Thrift et al. [29]	2.51 (1.63, 3.85)	2.08 (1.18, 3.69)				
Trivers et al. [23]	1.77 (0.93, 3.35)					
Wu et al. [32]		1.30 (1.01, 1.67)				
Jing et al. [25]	1.42 (0.83, 1.84)					
Lu et al. [24]	1.02 (0.61, 1.72)					
pooled estimates	1.27 (1.06, 1.53)	1.26(1.01, 1.60)				
EAC			/	/	0.0	0.960
Sundelof et al. [14]		1.00 (0.50, 1.70)				
Thrift et al. [31]		1.02 (0.64, 1.64)				
Trivers et al. [23]	1.08 (0.81, 1.44)					
pooled estimates		1.01 (0.70, 1.47)				

^a^*EC* esophageal cancer, *ESCC* esophageal squamous cell carcinoma, *EAC* esophageal adenocarcinoma, *HR* hazard ratio, *CI* confidence interval

^b^ The effect was estimated by comparing the highest with the lowest consumption of alcohol

**Fig. 4:**
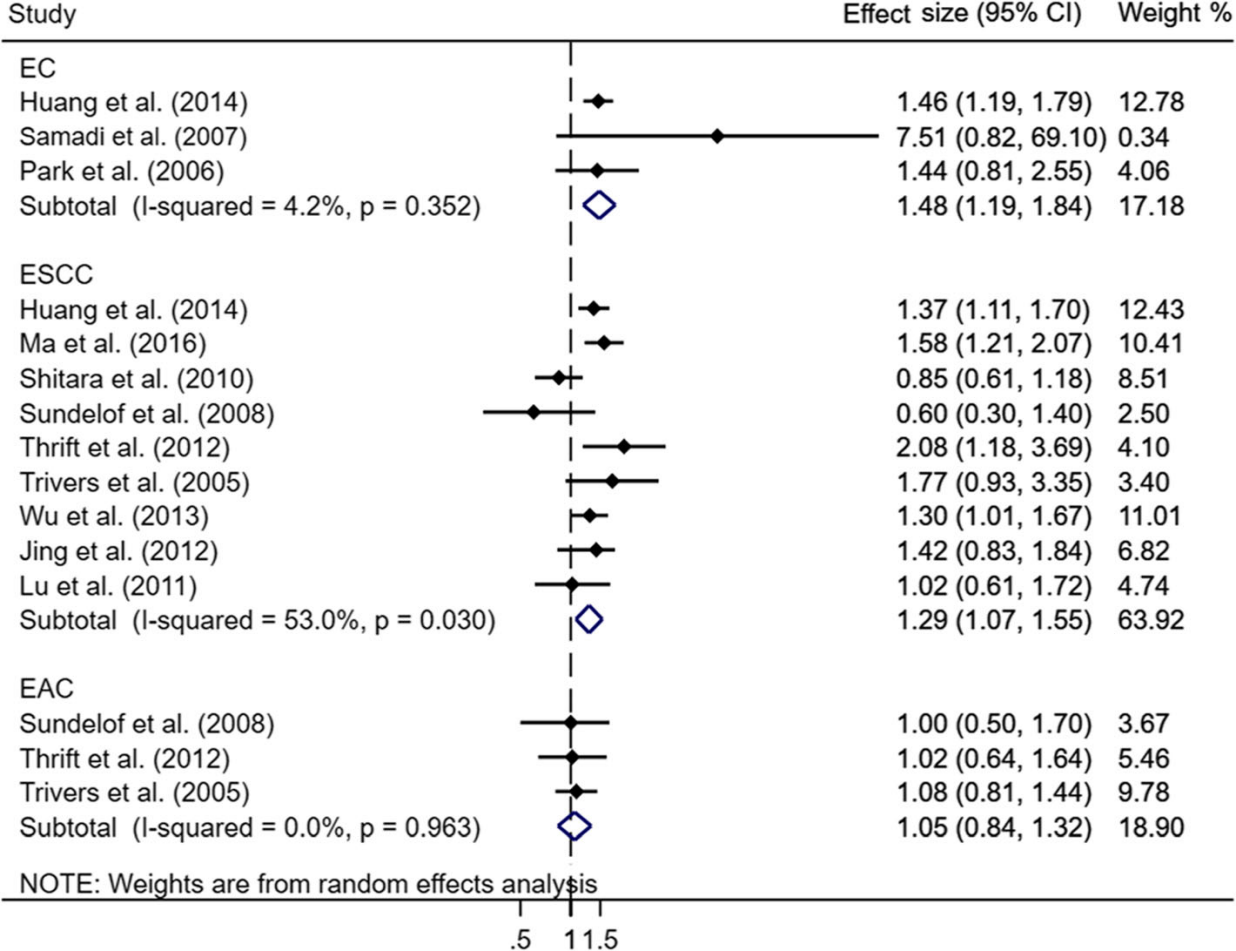
Forest plot of association between alcohol consumption (highest vs. lowest) and risk of mortality by cancer type. Abbreviation: EC, esophageal cancer; EAC, esophageal adenocarcinoma; ESCC, esophageal squamous cell carcinoma

**Fig. 5:**
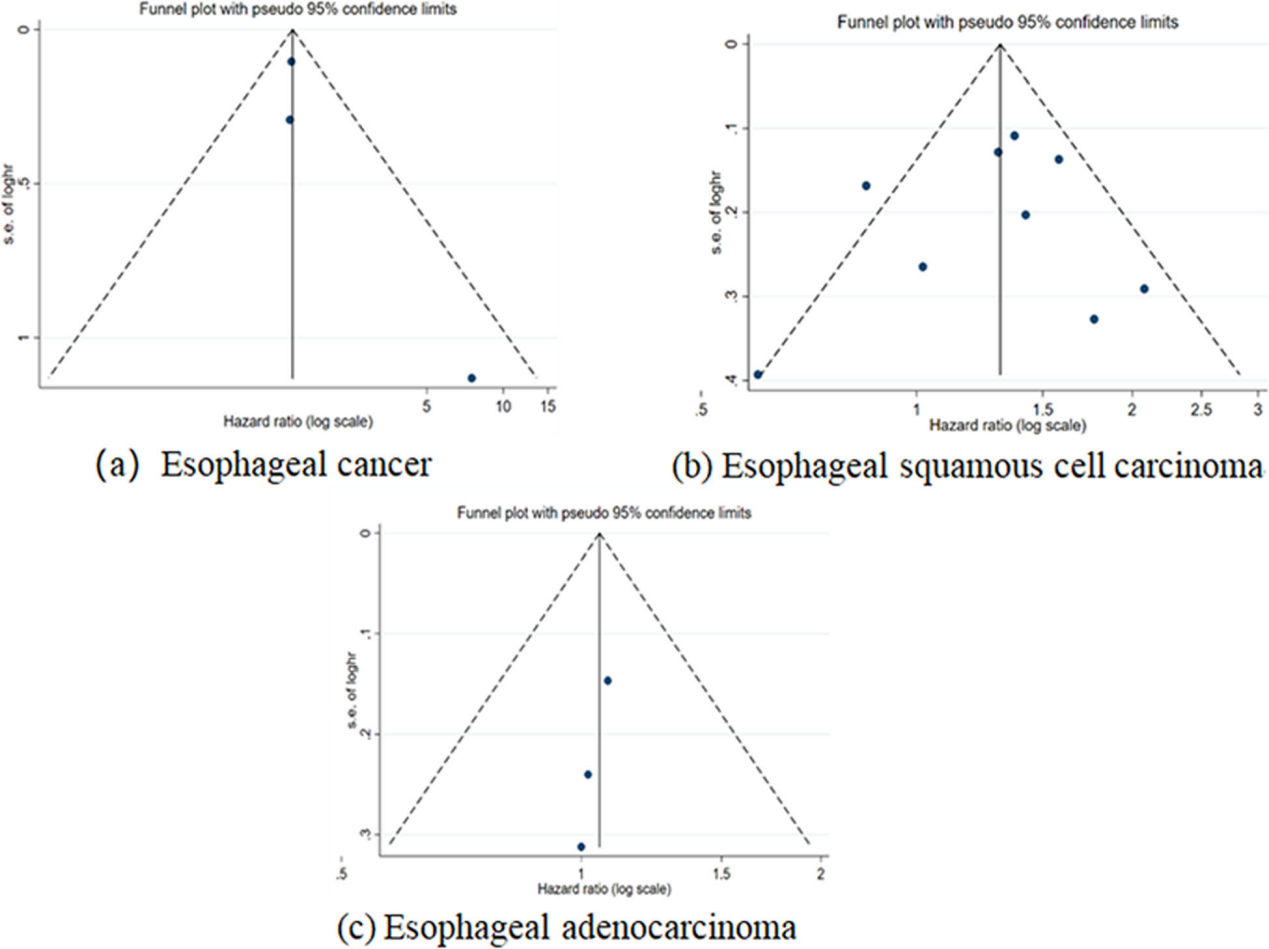
The funnel plot with Pseudo 95% confidence limits on alcohol consumption and risk of mortality by cancer type

The pooled results of the association between alcohol consumption and risk of death among different subtypes of esophageal cancer are shown in Fig. 4. When comparing the highest with lowest consumption of alcohol, the pooled HR was 1.48 (95% CI: 1.19–1.84) with low statistical heterogeneity (*P*_for heterogeneity_ = 0.35, *I*^2^ = 4.2%) in EC and 1.29 (95% CI: 1.07–1.55) with moderate statistical heterogeneity (*P*_for heterogeneity_ = 0.03, *I*^2^ = 53.0%) in ESCC, whereas no association was found in EAC (HR = 1.05, 95% CI: 0.84–1.32). The funnel plots did not show significant asymmetry for any types of esophageal cancer (Fig. 5), and the corresponding Begg’s tests did not show publication bias (all *P* > 0.30). Power calculation for these three random effect models was all equal to 100.0%.

The sensitivity analysis was only performed on alcohol consumption. The analysis was repeated consecutively by removing one study from the pooled results each time, and significant change was observed only after the exclusion of Huang’s study [27] in EC and ESCC, respectively (Supplementary Fig. S1); the analysis was also conducted by excluding 3 studies [23–25] not reporting adjusted effect size, and the pooled results were 1.26 (95% CI: 1.01–1.60) for ESCC and 1.01(95% CI: 0.70–1.47) for EAC (Table 2). When the unadjusted effect was considered, the pooled HR was 1.27 (95% CI: 1.06–1.53) for ESCC. Only one study [23] on EAC provided crude effect; hence, the pooled analysis was not conducted (Table 2). When the studies which did not include information of stage and severity of esophageal cancer were excluded, the pooled HR was 1.26 (95% CI: 1.01–1.60) for ESCC and 1.01(95% CI: 0.70–1.47) for EAC.

## Discussion

As far as we know, there lacks of systematic review with quantitative analysis to evaluate ordinary dietary behavior and prognosis of esophageal cancer. The results from meta-analysis displayed that pre-diagnostic dietary folate intake was significantly related to a decreased risk of esophageal cancer-specific mortality in ESCC and pre-diagnostic alcohol consumption was associated with an increased risk of all-cause mortality in EC and in ESCC.

Alcohol is one of the major determinant factors for developing esophageal cancer [34], but effects of it on esophageal cancer prognosis remain controversial [16]. The pooled results in our study showed that pre-diagnostic alcohol consumption could increase risk of mortality among EC and ESCC patients by 48 and 29% respectively; however, this effect was not found in EAC patients. Without adjustments, confounding factors could lead to misinterpretation of the association between independent variables and dependent variables [35]. Hence, we pooled crude HRs obtained from univariate analysis and adjusted HRs obtained from multivariate analysis, respectively; the association remained the same, further suggesting the strong effects of alcohol intake on ESCC mortality and indicating that our results was stable and robust. Similar results were obtained when the studies not including information of stage and severity of esophageal cancer were excluded. The findings from our study are consistent with the results from a previous meta-analysis by Fahey et al. [16], though the contrast groups were different between two studies. To examine the robustness of our results, we repeated the analysis by excluding the study one by one from the pooled results, and no significant change was observed for ESCC and EAC with the exception of excluding the study by Huang et al. [27], indicating the results our study obtained were stable. Omitting the study by Huang et al. [27] altered the positive association between alcohol consumption and risk of death in EC and ESCC to no association. This may be due to that the sample size of this study was the largest, therefore the weight of the study was relatively large when calculating the pooled results, indicating that more studies with large scale samples are needed.

Our results of meta-analysis also indicated that intake of dietary folate was associated with 59% reduced risk of esophageal cancer-specific mortality among ESCC patients. This finding is consistent with findings on other cancers [36, 37]. Folate metabolites have become diagnostic and therapeutic targets for several types of cancers in recent years [38, 39]. The possible mechanism maybe due to that folate deficiency indirectly affects DNA and RNA methylation, thereby alters the expression of tumor suppressor genes and proto-oncogenes [40, 41]. However, only two studies focused on effects of dietary folate intake on EC mortality, and all were carried out in China with small sample size. Thus, replication of our results in other large studies in different countries is warranted.

The evidence for other dietary components is limited. Lignans was found to reduce risk of all-cause mortality of ESCC by 42% [12], while positive association between the other two factors, sugary beverages and preserved vegetables, and esophageal cancer survival was revealed [13, 30]. Sugar in food can lead to awful disease progression through increased inflammation. The inflammation can be caused by oxidative stress, which ultimately accelerates DNA damage and elevates levels of interleukin-cytokines and other pro-inflammatory molecules [42]. N-nitroso compounds widely existing in processed foods (eg. preserved vegetables) may play an important role in tumor progression. N-nitroso compounds give rise to excessive expression of cyclinE 1, cyclinD 1, transform growth factor α and epidermal growth factor receptor in esophageal tissues, thus enhance cancer progression [43]. Anti-cancer effects of phytochemicals like lignans are mainly through estrogen/anti-estrogen activity, anti-proliferation or apoptosis, prevention of oxidation, induction of cell cycle arrest, regulation of changes in host immune system, anti-inflammatory activity and cell signal transduction [44].

This study has some strengths. First, the literature retrieval was from multiple databases and the selection was determined by independent reviewers, which was helpful to avoid literature omission. Second, we included studies according to the strict inclusion and exclusion criteria, which may augment the validity of our findings. Third, sensitivity analyses yielded similar results, indicating the stability of our findings.

There were also some limitations in this study. There were only 15 studies included; most of them focused on alcohol consumption and only five focused on other dietary components; pooling results from limited evidence may influence the stability of our results, though the power of random effect models in our study is fairly strong and sensitivity analyses yield similar results. Consuming large amounts of fruits and vegetables after diagnosis could reduce the mortality of cancer and diets before diagnosis may reflect the changes in taste or appetite attributable to cancer [45]. Besides, cancer patients may follow the doctor’s advice to change their diets in order to obtain better prognosis. However, dietary behaviors in this systematic review and meta-analysis were all pre-diagnostic, the roles of post-diagnostic dietary intake in prognosis of esophageal cancer are unclear.

## Conclusion

In summary, this review with limited evidence suggested that folate intake was associated with a reduced risk of esophageal cancer-specific mortality for ESCC, whereas alcohol consumption was associated with increased risk of mortality for ESCC. More studies are needed to investigate effect of dietary factors, especially post-diagnosis dietary consumption, on esophageal cancer prognosis.

## Supplementary information

**Additional file 1: Figure S1**. Summary of sensitivity analyses of alcohol consumption and mortality among (a) EC (b) ESCC (c) EAC. Abbreviation: HR, hazard ratio; CI, confidence interval; EC, esophageal cancer; EAC, esophageal adenocarcinoma; ESCC, esophageal squamous cell carcinoma.

## Data Availability

This study is a systematic review and meta-analysis, the data was extracted from published research. The data is available by contacting corresponding author or extracting from original published research.

## Abbreviations

CI: Confidence interval
EC: Esophageal cancer
ESCC: Esophageal squamous cell carcinoma
EAC: Esophageal adenocarcinoma
HR: Hazard ratio HR
NOS: Newcastle-Ottawa Scale
