# Supplementary material for: Dietary factors and risk of mortality among patients with esophageal cancer: a systematic review
Source: BMC Cancer. 2020 Apr 6;20:287. doi: 10.1186/s12885-020-06767-8 (PMC7137267; doi:10.1186/s12885-020-06767-8)
Supplement: Supplementary file 1 — Additional file 1: Figure S1. Summary of sensitivity analyses of alcohol consumption and mortality among (a) EC (b) ESCC (c) EAC. Abbreviation: HR, hazard ratio; CI, confidence interval; EC, esophageal cancer; EAC, esophageal adenocarcinoma; ESCC, esophageal squamous cell carcinoma. [file 12885_2020_6767_MOESM1_ESM.docx]

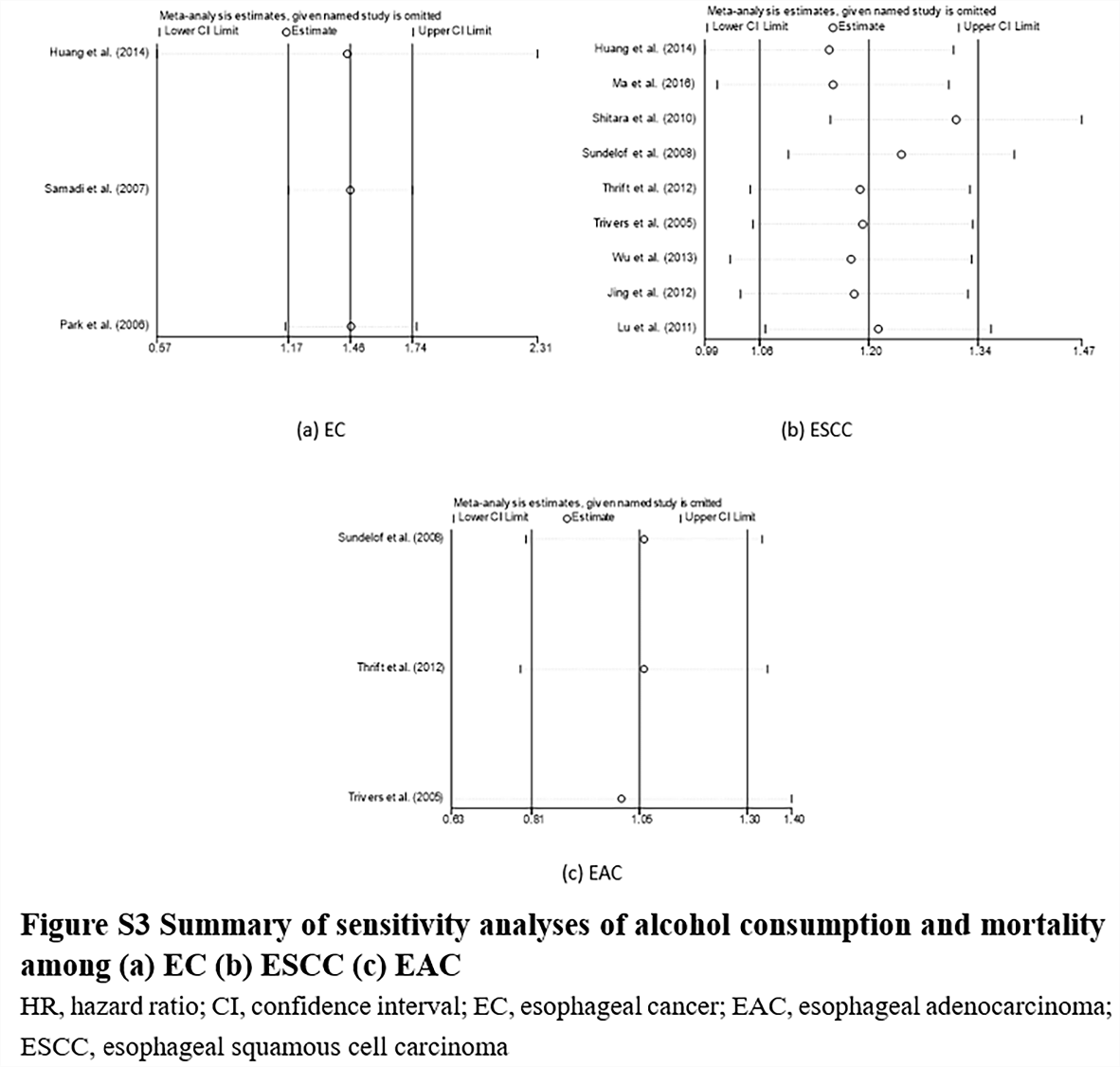


**Figure S1. Summary of sensitivity analyses of alcohol consumption and mortality among (a) EC (b) ESCC (c) EAC**

HR, hazard ratio; CI, confidence interval; EC, esophageal cancer; EAC, esophageal adenocarcinoma; ESCC, esophageal squamous cell carcinoma
